# Voting Systems for Environmental Decisions

**DOI:** 10.1111/cobi.12209

**Published:** 2014-01-01

**Authors:** MARK A BURGMAN, HELEN M REGAN, LYNN A MAGUIRE, MARK COLYVAN, JAMES JUSTUS, TARA G MARTIN, KRIS ROTHLEY

**Affiliations:** * Centre of Excellence for Biosecurity Risk Analysis, School of Botany, University of MelbourneParkville, 3010, Australia; † Biology Department, University of CaliforniaRiverside, CA, 92521, U.S.A.; ‡ Nicholas School of the Environment, Duke UniversityDurham, NC, 27708-0328, U.S.A.; § Department of Philosophy, Sydney Centre for the Foundations of Science, A14 Main Quadrangle, University of SydneySydney, NSW, 2006, Australia; ** Department of Philosophy, 151 Dodd Hall, Florida State UniversityTallahassee, FL, 32306-1500, U.S.A.; †† CSIRO Ecosystem Sciences41 Boggo Road, Dutton Park QLD 4102, Australia; ‡‡ Simon Fraser University, School of Resource and Environmental Management8888 University Drive, Burnaby, B.C., V5A 1S6, Canada

**Keywords:** Arrow’s theorem, decision theory, philosophy, preferences, Filosofía, preferencias, teorema de Arrow, teoría de decisión

## Abstract

Voting systems aggregate preferences efficiently and are often used for deciding conservation priorities. Desirable characteristics of voting systems include transitivity, completeness, and Pareto optimality, among others. Voting systems that are common and potentially useful for environmental decision making include simple majority, approval, and preferential voting. Unfortunately, no voting system can guarantee an outcome, while also satisfying a range of very reasonable performance criteria. Furthermore, voting methods may be manipulated by decision makers and strategic voters if they have knowledge of the voting patterns and alliances of others in the voting populations. The difficult properties of voting systems arise in routine decision making when there are multiple criteria and management alternatives. Because each method has flaws, we do not endorse one method. Instead, we urge organizers to be transparent about the properties of proposed voting systems and to offer participants the opportunity to approve the voting system as part of the ground rules for operation of a group.

Sistemas de Votación para Decisiones Ambientales

**Resumen:**

Los sistemas de votación agregan preferencias eficientemente y muy seguido se usan para decidir prioridades de conservación. Las características deseables de un sistema de votación incluyen la transitividad, lo completo que sean y la optimalidad de Pareto, entre otras. Los sistemas de votación que son comunes y potencialmente útiles para la toma de decisiones ambientales incluyen simple mayoría, aprobación y votación preferencial. Desafortunadamente, ningún sistema de votación puede garantizar un resultado y a la vez satisfacer un rango de criterios de desempeño muy razonable. Además, los métodos de votación pueden manipularse por los que toman las decisiones y votantes estratégicos si tienen el conocimiento de los patrones de votación y de las alianzas entre miembros dentro de las poblaciones votantes. Las propiedades difíciles de los sistemas de votación sobresalen en las tomas de decisiones rutinarias cuando hay criterios múltiples y alternativas de manejo. Ya que ambos métodos tienen fallas, no apoyamos a uno sobre el otro. En lugar de esto le pedimos urgentemente a los organizadores ser transparentes con respecto a las propiedades de los sistemas de votación y ofrecer a los participantes la oportunidad de aprobar el sistema de votación como parte de las reglas básicas para la operación de un grupo.

## Introduction

Environmental decisions lie at the interface of science and public policy. Scientists provide technical understanding, data, expert judgment, and predictions of the outcomes of alternative scenarios (Morgan & Henrion 1990; Cooke 1991; Burgman 2005; Patterson et al. 2007). Decisions also depend on the preferences of stakeholders: people interested in or affected by a decision. Stakeholders often have disparate values, interests, and prefer different tradeoffs among environmental, social, cultural, and economic criteria. Inevitably, differences of opinion arise (Slovic 1999; Maguire 2004). How should policy makers reconcile these divergent preferences to make decisions?

Voting is used widely to choose options, often because of its ease of application (e.g., Ghanbarpour et al. 2005). In conservation planning, it is used most often in informal settings to generate group preferences (e.g., Dicks et al. 2010). Despite its pervasiveness and practical appeal, there has been no systematic evaluation of voting for environmental applications. Importantly, different voting systems can generate different outcomes, even given the same underlying preferences. Yet, voting systems often are used unthinkingly and many people are unaware of the implications of choosing one voting system or another. The most familiar voting system allocates one vote per participant, and the alternative with the most votes is selected. However, this system has serious shortcomings, and there are many other voting systems with different and potentially more useful properties. We illustrate how different systems can generate different outcomes and describe the strengths and weaknesses of the alternatives, encouraging conservation biologists to think about desirable properties of different voting methods.

We examine how voting can be used to aggregate opinions about values and preferred strategies, rather than how voting can aggregate beliefs about facts. We assume that participants (voters) and the alternatives are known; group member selection and identification of stakeholders and feasible alternatives have been discussed elsewhere (e.g., Keeney 1992; Burgman 2005; Burgman et al. 2011). We also ignore the many psychological factors that influence group decisions (Slovic 1999; Kerr & Tindale 2004).

This review focuses on a subset of voting systems with the greatest potential utility for environmental decision making. We describe simple majority, approval voting, and preferential voting and outline 2 approaches with very long histories of application, the Borda count and Condorcet functions. We evaluate the mathematical and cognitive properties of these systems, identify circumstances in which voting system attributes are likely to be important, and describe how voters may manipulate systems. We conclude with recommendations for using voting systems for environmental decisions.

## Methods

We searched the ISI Web of Science from 1990 to 2012 for papers describing voting methods using “voting,” “preference aggregation,” and “group decision.” We used terms discovered in this initial search (*decision*, *environment*, *conservation*, *group*, *vote*, and *preference*) to find applications of voting methods in ecology, conservation biology, and environmental science. References in these papers were used to identify additional studies that applied a voting method to aggregate individual preferences for environmental issues. In total we found 21 articles that used at least one of the voting methods focused on in this study or that used a variation of them that was relevant or that illustrated their use. We cite and discuss the applications in the relevant sections of the review.

### Concepts and Notation

We devised a formal notation for the voting systems described below. Group decisions involve sets of individuals, *I*, alternatives, *A*, and preferences, *P*. We let *I* = {*I_i_* : *i* = 1, 2, …, *m*} be the set of *m* individuals, *A* = {*A_j_* : *j* = 1, 2, …, *n*} be the set of *n* alternatives, and *P* = {*P_i_* : *i* = 1, 2, …, *m*} be the set of preferences of the individuals in *I*, where *P_i_* is a set of preferences over *A* for individual *I_i_*. Voting methods for group decisions involve evaluating the set of alternatives, *A*, on the basis of individual preferences, *P*.

A group evaluation takes the form of an ordering, *R*, of alternatives which indicates, for each pair of alternatives *A_j_*, *A_k_*, whether the group prefers *A_j_* to *A_k_*, prefers *A_k_* to *A_j_*, or is indifferent between the two. Group decision methods thus define a function, *W*, on *P* such that *W*(*P*) = *R*. Following Arrow (1951), *W* is described as a social welfare function. For the purposes of this review, a voting method combines a protocol for determining individual preferences with a social welfare function *W* to identify the set of alternatives the group prefers to all others, from the set of individual preferences, *P*. We ignore quantitative information and focus solely on the ordinal preference information of *P* to identify that set of alternatives. We assume initially that *P* includes enough information about preferences of individuals in *I* to define a preference relation, ≻*i*, on *A* for each individual *I_i_* ∈ *I*, where the alternative *A_j_* ≻*i A_k_* if and only if individual *I_i_* prefers *A_j_* to *A_k_*.

Let ≻*i* represent “strict preference” and define ≥*i* as the “weak preference” of *I_i_* for *A_j_* over *A_k_* (i.e., where *A_j_* ≥*i A_k_* means individual *I_i_* prefers or is indifferent to *A_j_* over *A_k_*) and ∼*i* as “indifference” (i.e., where *A_j_* ∼*i A_k_* means individual *I_i_* is indifferent between them).

Ordinal preferences refer to ranks of preferences. A preference relation ≻*i* defines an ordinal value function *O_i_*: *A* → *R* on *A* where *O_i_*(*A_j_*) > *O_i_*(*A_k_*) if and only if *A_j_* ≻*i A_k_*. The *O* therefore indicates the results of pairwise comparisons of alternatives, that is, whether *A_j_* ≻*i A_k_*, *A_k_* ≻*i A_i_*, or *A_i_* ∼*i A_k_*.

### Properties of an ideal system

Different ways of considering preferences can lead to different decisions. For example, consider 7 stakeholders who must decide which of 3 environmental strategies is best. The alternatives are *A*_1_ maximize harvest, *A*_2_ protect threatened species, and *A*_3_ maximize public amenity. Three stakeholders prefer *A*_1_ to *A*_2_ and *A*_2_ to *A*_3_ (i.e., *A*_1_ > *A*_2_ > *A*_3_). Two stakeholders prefer *A*_2_ to *A*_1_ and *A*_1_ to *A*_3_ (i.e., *A*_2_ > *A*_1_ > *A*_3_). Two prefer *A*_3_ to *A*_2_ and *A*_2_ to *A*_1_ (i.e., *A*_3_ > *A*_2_ > *A*_1_). Alternative *A*_1_ gets 3 first place votes, and each of the other alternatives only gets 2. But a clear majority (4 of 7) prefers *A*_2_ to *A*_1_. Which alternative should the policy maker select?

Three appealing properties of any voting system are transitivity, completeness, and the Pareto principle.

1. Transitivity requires that if an individual or the group prefers *A*_1_ over *A*_2_ (*O*(*A*_1_) ≻ *O*(*A*_2_)) and *A*_2_ over *A*_3_ (*O*(*A*_1_) ≻ *O*(*A*_3_)), then they prefer *A*_1_ over *A*_3_ (*O*(*A*_1_) ≻ *O*(*A*_3_)). Consider a group that prefers to maximize harvest to protect threatened species and prefers to protect threatened species over maximizing public amenity. If the group is given the choice to maximize harvest or public amenity, transitivity means they would prefer to maximize harvest.
2. The Pareto principle states that if *A*_1_ is preferred to *A*_2_ by all individuals, then the group should also prefer *A*_1_ over *A*_2_. More generally, Pareto efficiency (or Pareto optimality) means that if the group preference is *A*_1_ ≻ *A*_2_, it implies *A*_1_ is better than *A*_2_ from the perspective of at least one person and no one regards it as worse. Thus, if protecting public amenity in the example above is the least preferred of the 3 alternatives for all individuals, then the voting system should not select it.
3. Completeness requires the voting system give a group preference for all of the alternatives in *A*.

There are other appealing properties of voting systems. For example, we may wish to select the alternative that would defeat every other alternative in one-on-one (pairwise) comparisons. Other desirable properties may be social or psychological. For example, it may be important that all members of the group understand how the voting system works (Elkind et al. 2011). Others writing about desirable properties of voting systems (Armbruster & Boge 1983; Fishburn & Brams 1983; Hwang & Lin 1987; Saari 2001; Hodge & Klima 2005; Poundstone 2008; Elkind et al. 2011) have proposed the following:

1. Homogeneity: The result of a vote depends on the proportions of the total number of votes assigned to each alternative, and not on their absolute counts.
2. Monotonicity: Increasing the number of votes for a winning alternative cannot make it a loser, and a losing alternative cannot become a winner when its number of votes decreases.
3. Anonymity: Voters are treated the same in the sense that if any 2 individuals trade their votes, the outcome would be the same.
4. Decisiveness: The voting system delivers an unambiguous winner or a winning set, if more than one alternative is desired.
5. Consistency: If a group is divided into subgroups and each subgroup selects the same alternative, then the entire group also selects that alternative.
6. Invulnerability to the no-show paradox: It is not possible for a group of voters to obtain their first-ranked choice by abstaining if voting would lead to the selection of some other option.

Although each of these properties seems intuitively desirable, some have several interpretations, some are inconsequential in many situations, some are mutually exclusive and others have undesirable implications in some circumstances.

Arrow (1951) proved that when 3 or more alternatives exist, no voting system can convert voter preferences into a Pareto efficient, complete, and transitive rank while satisfying these very reasonable criteria:

1. If every individual prefers *A*_1_ to *A*_2_, then the group should prefer *A*_1_ to *A*_2._
2. There should be no individual in the group who can determine the group’s preferences (no dictatorship). More formally, there is no individual *i* such that *A*_1_ ≻ *A*_2_ implies the social choice of *A*_1_ over *A*_2_ regardless of the preferences of all other individuals.
3. The solution should be independent of irrelevant alternatives. That is, the ranking of *A*_1_ relative to *A*_2_ should not depend upon the introduction of another alternative, *A*_3_.

The importance of these and other intuitively desirable properties depends on the context in which a voting system is applied. For example, when choosing one alternative, completeness may not be important. Votes may be used to choose a subset of acceptable alternatives, unranked, in which case resolving all ties is not essential. Anonymity may not be desired when votes are deliberately weighted unequally, reflecting, for example, the number of shares individuals hold in a public company. Some voting systems may not guarantee a clear winner, but if there are many more individuals than alternatives, an indecisive outcome may be very unlikely.

## Voting Systems

Return to the hypothetical example outlined above in which a group of stakeholders is asked for their preferences for 3 mutually exclusive environmental strategies, each of which has a different focus: *A*_1_ maximize harvest, *A*_2_ protect threatened species, and *A*_3_ maximize public amenity. The preferences expressed by 2 groups of 3 individuals (*I*) are arranged in Table1.

**Table 1:** Hypothetical ordinal preferences of 2 groups (a, b) of 2 individuals (*I*) for 3 alternatives (*A*)

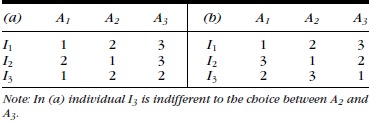

### Removing Dominated Alternatives

The simplest defensible action a decision maker can make is removing “dominated” alternatives. In general, dominated alternatives are not the favorite of any individual. Therefore, in Table1a, the alternative set is reduced to *A*_1_ and *A*_2_. However, this does not provide a complete ranking of the remaining alternatives, *A*_1_ and *A*_2_, which are Pareto efficient alternatives (i.e., they are preferred by at least one individual).

This approach (removing dominated alternatives) is used in multicriteria decision analysis to simplify problems (e.g., Gregory & Keeney 2002). Hämäläinen et al. (2001) used it to analyze 5 water level management plans for a lake-river system in Finland involving 10 groups of stakeholders, including power producers, farmers, environmentalists, recreational users, and fishers and 10 ecological, economic, and sociological criteria.

### Simple Majority (Plurality) Voting

Under simple majority voting, each voter has one vote, which may be cast for a single alternative. Thus, stakeholders cannot express their preferences for all of the alternatives; they merely identify their best option. Under simple majority voting, the aggregation rule is to sum the votes for each alternative. The alternative with the most votes is selected. There is no requirement that an alternative acquire a majority (>50%) of votes.

In antiplurality voting, individuals apply a single veto. The alternative with the largest number of vetoes is removed. The set of alternatives may be reduced to a desired size through successive vetoes (Armbruster & Boge 1983).

Consider the preferences of 9 individuals for 3 alternatives (Table2). Under simple majority voting, *A*_1_ would be selected, even though 5 of the 9 individuals prefer either *A*_2_ or *A*_3_ to *A*_1_. Thus, the preference ordering suggested by the count of votes in Table1 may not capture the full set of individual preferences and may result in unsatisfactory choices from the perspective of the majority. Below, we discuss how this property may be exploited to deliver outcomes at odds with the preferences of the majority of voters.

**Table 2:** Hypothetical ordinal preferences of 9 individuals (*I*) for 3 alternatives (*A*)

	*A*_1_	*A*_2_	*A*_3_
*I*_1_	1	2	3
*I*_2_	1	2	3
*I*_3_	1	2	3
*I*_4_	1	3	2
*I*_5_	3	2	1
*I*_6_	3	2	1
*I*_7_	3	2	1
*I*_8_	3	1	2
*I*_9_	3	1	2
**Votes**	**4**	**2**	**3**

Above we noted that transitivity is a desirable property of a voting system. Unfortunately, if more than 2 alternatives exist, simple majority voting may not be transitive (Arrow 1951; Dodgson 1876; Gehrlein 1983). For example, consider the case in Table1b, where 3 individuals specify their preferences for the 3 alternatives.

For *A*_1_ and *A*_2_, *O_i_*(*A*_1_) > *O_i_*(*A*_2_) holds for 2 individuals and *O_i_*(*A*_2_) > *O_i_*(*A*_1_) holds for one individual. Thus, *R* = (*A*_1_ > *A*_2_).

For *A*_2_ and *A*_3_, *O_i_*(*A*_2_) > *O_i_*(*A*_3_) holds for 2 individuals and *O_i_*(*A*_3_) > *O_i_*(*A*_2_) holds for one individual. Thus, *R* = (*A*_2_ > *A*_3_).

For *A*_1_ and *A*_3_, *O_i_*(*A*_1_) > *O_i_*(*A*_3_) holds for one individual and *O_i_*(*A*_3_) > *O_i_*(*A*_1_) holds for 2 individuals. Thus, *R* = (*A*_3_ > *A*_1_).

Thus, for this set, *R* is not transitive.

Another drawback of simple majority voting is that the preference of the group for one of 2 alternatives can be influenced by the inclusion or exclusion of other alternatives. For example, if we were to remove *A*_3_ from Table2 and reassign the preferences, then *A*_1_ would have 4 votes and *A*_2_ would have 5. The votes for *A*_2_ have been effectively split between *A*_2_ and *A*_3_. If every choice involved only 2 alternatives, then the outcome could be determined equitably and consistently through simple majority voting. If there are many alternatives, the winner may attract only a small percentage of total votes cast, which may be unsatisfying even if it does not violate any of the other desirable qualities listed above.

Given the potential frailties of simple majority voting, it is perhaps surprising how widely it is used to make environmental management decisions. Examples include nomenclatural decisions in taxonomy (e.g., McNeill & Turland 2010). (Although, interestingly, for the naming of *Acacia*, a 60% majority threshold was required.) In environmental management it has been used to identify a preferred management strategy for threatened species (Marcot et al. 2006) and to identify preferred forest management options (Kangas et al. 2006; Hiltunen et al. 2008). Tisdell et al. (2006) used it to assess preferences for conservation objectives for Australian vertebrates. In Chile, members of the National Fisheries Council vote on proposals for total allowable catch by a simple majority vote (Leal et al. 2010).

### Approval Voting

In approval voting (Brams 2004) individuals select all alternatives of which they approve. The alternative(s) with the most votes is (are) selected. Generally, the option with the greatest overall support attracts the largest number of votes (Brams 2004). To illustrate its properties, we use Table2 and specify that the 9 individuals who expressed preferences would approve of their first and second choices but not their third. Other configurations of approvals would lead to different results. Their approval votes would be as in Table3.

**Table 3:** Hypothetical approval votes cast by the 9 individuals (*I*) in Table2 for alternatives (*A*)

	*A*_1_	*A*_2_	*A*_3_
*I*_1_	1	1	
*I*_2_	1	1	
*I*_3_	1	1	
*I*_4_	1		1
*I*_5_		1	1
*I*_6_		1	1
*I*_7_		1	1
*I*_8_		1	1
*I*_9_		1	1
**Votes**	**4**	**8**	**6**

In this example, *A*_2_ would be selected. Approval voting has the advantage over simple majority voting in that its results are transitive and it captures more information about the preference set *P*. However, it can generate counter-intuitive outcomes from the perspective of the choice of a single, most-preferred alternative. In this example, *A*_2_ was selected even though the fewest voters preferred it (Table2). If a second alternative were selected based on the approval vote, it would be *A*_3_, even though most voters preferred *A*_1_. Nevertheless, most voters considered *A*_2_ to be good enough. Approval voting tends to promote moderate alternatives that may be satisfactory but not ideal from any one perspective (Kangas & Kangas 2002).

Approval voting is used widely to identify group preferences in ecology and conservation biology. Sutherland et al. (2009) (see also Morton et al. 2009) used Approval voting to reduce a long list of global conservation questions to a more manageable list. In Brown et al. (2010), each participant allocated 10 votes among 94 water research questions, and the questions with the most votes were selected, creating a priority list of 15 questions. Vignola et al. (2012) used it to identify acceptable alternatives for the management of a catchment, reconciling the priorities of upstream farmers with downstream hydropower producers. Kangas et al. (2006) reviewed its application to choices in forest management.

### Preferential Voting

Preferential voting and the Borda count (following) use the entire preference order to determine *R*. Preferential voting (also called alternative vote or instant run-off) is designed to give equitable consideration to the full range of each voter’s preferences. Under this system, if an alternative receives sufficient first preferences to achieve a quota (i.e., a minimum number of votes), it is selected. If no alternative achieves sufficient votes, the votes allocated to the least popular alternative are redistributed (transferred) to the remaining alternatives with the following procedure. Remove the alternative with the fewest primary votes. If there is a tie, remove the alternative with the fewest second preferences among those that tie. If there is a second tie, remove the alternative with the fewest third preferences among those that tie, and so on.

When there are *m* voters and more than one alternative is to be selected, transfers are made sequentially until *n* alternatives attain the quota needed for election. The quota, *Q*, is usually defined as (Fishburn & Brams 1983),

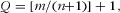
1where the brackets indicate the integer part of the argument.

In Table4, all 3 alternatives are scored first once, but *A*_3_ is never scored second, so it is removed. Of the remaining alternatives, *A*_1_ and *A*_2_, *A*_2_ has the fewest first preferences so it is removed. Thus, *R* = (*A*_1_ > *A*_2_ > *A*_3_).

**Table 4:** Hypothetical ordinal preferences of 3 individuals (*I*) for 3 alternatives (*A*) after the first round of voting and the second round of voting when preferences delineated in the first round are applied

First round				Second round	
	*A*_1_	*A*_2_	*A*_3_	*A*_1_	*A*_2_
*I*_1_	1	2	3	1	2
*I*_2_	2	3	1	1	2
*I*_3_	2	1	3	2	1

In preferential voting, an alternative that would attract the most votes in simple majority voting or an alternative that can defeat every other alternative in direct-comparison (one-on-one) voting (i.e., the Condorcet winner; see below) might not be selected (Fishburn & Brams 1983). Preferential voting may violate the monotonicity criterion, such that an increase in support for an alternative may turn it from a winner into a loser. In Table5 decision 1, the fewest people prefer option *A*_2_. It so happens that all of the voters who prefer *A*_2_ also prefer *A*_3_ over *A*_1_. Their votes in round 2 transfer to option *A*_3_, which is selected.

**Table 5:** Example of preferential voting showing how an increase in support for an alternative can turn it from a winner into a loser. Numbers in the table are the number of votes received for each alternative

	*A*_1_	*A*_2_	*A*_1_
***Decision 1***			
Round 1	11	8	10
Round 2	11		18
***Decision 2***			
Round 1	7	8	14
Round 2		15	14

Note The only change between decision 1 and decision 2 is that in Decision 2, support for *A*_3_ increases in the form of 4 votes moving from *A*_1_ to *A*_3_.

In decision 2, the only change is that support for *A*_3_ increases, with 4 votes being transferred from *A*_1_ to *A*_3_. As a result, *A*_1_ is removed first. It so happens that all of the voters who prefer *A*_1_ also prefer *A*_2_ over *A*_3_. The preferences associated with *A*_1_ transfer to *A*_2_, which is selected over *A*_3_. The outcome is that *A*_3_ is no longer selected even though support for it increased. Although this counter-intuitive outcome is possible, it required quite an unusual distribution of preferences (namely, all voters who prefer *A*_2_ also prefer *A*_3_ over *A*_1_ and all voters who prefer *A*_1_ also prefer *A*_2_ over *A*_3_). This raises the question of whether such arrangements of preferences are likely to arise in practice. We explore this question below.

Despite the appeal of systems that incorporate each individual’s full set of preferences, we could find no examples of preferential voting in environmental science or conservation decision making. It is implemented in many jurisdictions in Australia to choose political representatives and has affected the development of public environmental policy there (Williams 2006).

### The Borda Count

Like preferential voting, the Borda count uses the entire preference order of participants, but instead of reallocating the votes as is done under preferential voting, the preferences are tallied. When there are *n* alternatives, the best alternative gets *n* – 1 points, the second *n* – 2 points, the third *n* – 3 points, and so on. The least preferred option scores zero. The Borda counts for preferences shown in Table4 are given in Table6. The Borda tally aggregates individual preferences to generate the group’s preferences. The tallies in Table6 suggest the ranks *R* = (*A*_1_ > *A*_2_ > *A*_3_).

**Table 6:** Hypothetical Borda counts for 3 individuals (*I*) for 3 alternatives (*A*) applied to the preferences in Table4

	*A*_1_	*A*_2_	*A*_3_
*I*_1_	2	1	0
*I*_2_	1	0	2
*I*_3_	1	2	0
Tally	4	3	2

The Borda count has been used to aggregate preferences in many contexts (Black 1958; Hwang & Lin 1987). Kijazi and Kant (2010) used a Borda count to establish group preferences for alternatives for forest use on Mount Kilimanjaro. Laukkanen et al. (2005) and Hiltunen et al. (2008) evaluated group preferences for forest management plans in Finland, applying several voting methods including the Borda count. Laukkanen et al. (2005) asked individuals to provide an ordering of forest plans on each of several criteria with approval voting and then used the Borda count to combine individual preferences into a group ordering.

### Condorcet Functions

A Condorcet winner has the appealing property that it is preferred to any other alternative by a majority of the voters (see Elkind et al. 2011). Condorcet functions find the preferred alternative by examining all pairwise comparisons of alternatives for each individual (a total of *n* × (*n* – 1)/2 comparisons for *n* alternatives). Each individual votes on each comparison. The score for an alternative is given by the number of times it is ranked higher than another. That is, *O_i_*(*A_j_*, *A_k_*) = 1 if and only if *A_j_* ≻*i A_k_* and *O*(*A_j_*) is summed over *n* alternatives and *m* individuals:

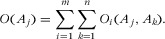
2

For example, Table7 gives the preferences of 4 individuals for 3 alternatives. The resulting table of ordinal preferences, *O*, and resulting Condorcet scores are given in Table8. The *O*(*A*_1_) is the sum of columns *O*(*A*_1_, *A*_2_), and *O*(*A*_1_, *A*_3_) = 6, *O*(*A*_2_) = 2, and *O*(*A*_3_) = 3 so that *R* = (*A*_1_ > *A*_3_ > *A*_2_).

**Table 7:** Hypothetical ordinal preferences of 4 individuals (*I*) for 3 alternatives (*A*)

	*A*_1_	*A*_2_	*A*_3_
*I*_1_	1	3	2
*I*_2_	1	3	2
*I*_3_	3	1	3
*I*_4_	1	3	2

**Table 8:** Condorcet scores for pairwise comparisons of alternatives in Table7

	*O*	*O*	*O*	*O*	*O*	*O*
	(*A*_1_, *A*_2_)	(*A*_1_, *A*_3_)	(*A*_2_, *A*_1_)	(*A*_2_, *A*_3_)	(*A*_3_, *A*_1_)	(*A*_3_, *A*_2_)
*I*_1_	1	1	0	0	0	1
*I*_2_	1	1	0	0	0	1
*I*_3_	0	0	1	1	0	0
*I*_4_	1	1	0	0	0	1
**Tallies**	**3**	**3**	**1**	**1**	**0**	**3**

Ghanbarpour et al. (2005) and Zendehdel et al. (2010) used a Condorcet function to aggregate group opinions about management alternatives for a watershed in Iran. Pairwise procedures such as the Condorcet function ignore portions of the information that are used by other procedures such as the Borda count and preferential voting. Of course, simple majority voting ignores even more.

### Other Functions

Many other voting systems have been developed. They have not been used widely for environmental decision making, but we document them so that readers may pursue them if they require a particular feature for a specific context.

Variants of the Condorcet function, including Copeland’s function (Hwang & Lin 1987), select alternatives based on pairwise comparisons, their differences depending on the form of the comparisons (e.g., Craven 1992; Risse 2001). Dodgson’s (1876) function orders alternatives according to the number of pairwise comparisons that would require alteration for an alternative to be preferred to all others (Hwang & Lin 1987; Elkind et al. 2011).

In the Hare system, sometimes called 2-stage plurality, individuals cast one vote for one alternative. These are tabulated and the alternative with the smallest tally is removed from *A*. This process is repeated until one alternative remains. Hare voting requires that votes be tallied and results disseminated before another round of voting can take place, significantly increasing the complexity of the voting process.

Nanson’s (1883) function identifies preferences through iterative application of the Borda count (Hwang & Lin 1987). Alternatives with the lowest Borda count are sequentially eliminated until a set of alternatives with the same Borda count remains. Group preferences derived from Nanson’s function may differ from those derived from simple Borda counts.

Fishburn’s (1977) function gives a group preference *A*_1_ > *A*_2_ if and only if *A*_1_ outscores or ties *A*_2_ by simple majority and *A*_1_ outscores or ties at least one other alternative that outscores *A*_2_. Kemeny’s (1959) function scores alternatives by the number of times each is ranked higher than another. Its goal is to maximize the agreement between final group preferences and individual voters’ preferences; matrix algebra is used to find a solution (Fishburn 1977; Hwang & Lin 1987). It is considered far too complex for many voters to understand.

Some systems allocate multiple votes to each individual. If individuals may allocate multiple votes to a single alternative if they strongly prefer it (e.g., Mendoza & Prabhu 2009), the method is equivalent to assigning weights rather than voting. This is sometimes called cumulative voting. New voting systems continue to be developed. The examples outlined here illustrate the richness of potential approaches.

Individual preferences can be expressed by the strength of preferences among alternatives (sometimes called cardinal data). Data on preference strengths can be collected via focus groups, deliberative multicriteria analysis, or remotely via surveys of stated preferences or willingness to pay (e.g., Saaty 1980; Gregory & Keeney 2002; Chee 2004). These methods often require travel and face-to-face workshops, extensive sampling protocols, or complex question formats. Cost and fatigue can make these systems impractical.

### Do the Differences Matter?

As illustrated above, counter-intuitive and even unintended outcomes are possible when voting methods are applied unthinkingly. Riker (1982), Poundstone (2008), and others argue that because all voting procedures have at least one serious flaw and opportunities for strategic manipulation are ubiquitous, voting results cannot express collective opinion. The list of important properties of voting systems in Table9 is incomplete, but even so, no method satisfies them all.

**Table 9:** Flaws of voting systems (modified from Nurmi 2012; see Richelson 1981)

Voting system[Table-fn tf9-1]					
	Simple				
Criteria	Majority	Approval	Preference	Borda	Condorcet
Condorcet winner is chosen	0	0	0	0	1
Monotonicity	1	1	0	1	1
Pareto optimality	1	0	1	1	1
Consistentency	1	1	0	1	0
Independence[Table-fn tf9-2]	0	0	0	0	0
Invulnerability[Table-fn tf9-3]	1	1	0	1	0
Majority winner	1	0	1	0	1

a Numbers: 1, system satisfies the criterion; 0, system violates criterion.

b Independence of irrelevant alternatives.

c Invulnerability to no-show paradox.

Arrow’s theorem applies to open and secret ballots, even when voters are unaware they are voting, such as when they reveal preferences through their purchases (French 1986). Saari (2001) notes that the same problems arise when individuals make choices based on a number of independent and incommensurate criteria, as is commonplace in conservation management. Consider an example in which a manager is required to select a conservation reserve from among 4 areas that contain 4 threatened species, 3 threatened communities, and 4 important ecosystem services. A field survey results in the data in Table10.

**Table 10:** Hypothetical measures for each of 11 criteria for 4 candidate conservation reserves (based on tables 1.1 and 1.2 in Saari 2001)

Measure	Area A	Area B	Area C	Area D
Populations of threatened species 1	0	20	10	80
Populations of threatened species 2	4	0	3	2
Populations of threatened species 3	30	20	18	12
Populations of threatened species 4	2	9	8	15
Extent of threatened ecosystem 1	400	50	80	100
Extent of threatened ecosystem 2	7	0	2	3
Extent of threatened ecosystem 3	0	25	30	10
Index of water quality	3	7	8	5
Index of cultural value	4	8	6	10
Index of pollination services	44	80	60	100
Tourism revenue	8	5	2	1

Note Units of measure: threatened species, number of adults; ecosystem extent, hectares; ecosystem services, constructed scales; tourism revenue, millions of dollars per annum. The units are omitted on the table to emphasize interest only in rank of each option for each criterion.

The scores for the 11 criteria generate a rank order for the manager’s preference for each reserve. There is no clear winner over all criteria, and no clear loser, so the manager cannot eliminate any dominated alternatives. A natural approach is to select the ‘best of the best’ (Saari, 2001), where the manager finds the top-ranked alternative for each criterion and selects the option ranked best most often. This is equivalent to plurality voting.

The manager’s objective may be to avoid making a bad choice on any of the criteria. One way of doing so awards votes to an option if it is either first or second on each criterion. This is equivalent to approval voting. Alternatively, the manager may wish to account for all attributes in a balanced fashion, awarding 3 votes to the best option, 2 to the second best option, and 1 to the third best option for each criterion. This is equivalent to Borda count. In this hypothetical but quite realistic example, area A is chosen under a plurality vote, area B is chosen under an approval vote, and area D is chosen under a Borda count or preferential vote.

There are several problems with Table9 as a basis for choosing a voting system. First, it is not comprehensive about what is good in a system. Some of these other attributes have been outlined in the preceding discussion. Second, the criteria are not equally important or independent. For example, the Condorcet winner criterion is strictly incompatible with the consistency criterion (Young 1974 in Nurmi 2012). Perhaps most importantly, the *potential* for violations of the criteria says nothing about how likely these violations are to arise in particular circumstances in which the system is used.

Assessing the likelihood of violations can be difficult because it relies on assumptions about the distributions and independence of preferences (see Nurmi 2012). In general, though, given strong preferences (full ordering of preferences), a small numbers of voters (less than about 10) or many alternatives (more than about 10), as might happen in a small committee, it is likely the Condorcet system will not produce a preferred alternative. The probabilities of intransitive outcomes are even greater than failing to find a Condorcet winner (Klahr 1966; Jones et al. 1995). In contrast, such problems are unlikely to arise if there are many voters with weak preferences (such that individuals may be indifferent to some alternatives), few alternatives (Jones et al. 1995), a few relatively strong candidates (Tangian 2000), or coalitions of voters with similar preferences (Nurmi 2012).

All voting rules are susceptible to strategic manipulation to greater or lesser degrees (Satterthwaite 1975). People designing voting systems may manipulate outcomes by including alternatives so that votes for a preferred alternative are split among 2 or more similar alternatives (Gibbard 1973). It may be in the best interests of a voter to vote for an alternative other than the one they prefer most, or not to vote at all, to secure their desired result (Kangas & Kangas 2002). A major drawback of simple majority voting, for example, is that it is relatively easy to manipulate by introducing irrelevant alternatives (Lehtinen 2008). Consider the preferences in Table11 (derived from Table2). Under simple majority voting, *A*_2_ is selected (decision 1). A person who controls the design of the voting system may have a personal preference for *A*_1_. If they know the likely distribution of votes among the alternatives, they may add another alternative with attributes similar to *A*_2_, in which case *A*_1_ would be selected if votes were now distributed evenly across *A*_2_ and the new alternative (decision 2).

**Table 11:** An example of a strategic split of votes

	*A*_1_	*A*_2_	*A*_3_
Decision 1	4	**5**	
Decision 2	**4**	2	3

Strategic voting and coalitions have been explored at length in political science (see Taylor 1995; Hodge & Klima 2005). Generally, manipulation requires the system designer or the voter to know about other voters’ preferences. Voters with more information have more power. For instance, approval voting is more difficult to manipulate than simple majority voting because it requires additional information about the distribution of approvals (Lepelley & Valognes 2003). Thomas et al. (2010) showed how strategic voting behavior may evolve over time into special interest-driven voting blocks that “capture” state agencies, leading to decisions about the environment by public regulatory agencies that accord with commercial or recreational interests.

## Discussion

Many decisions in conservation biology and environmental science involve reconciling disparate, competing objectives and thus may be viewed as attempts to maximize net social welfare. We described the characteristics of some common voting systems and outlined examples from environmental science and conservation biology. Most of the application papers that we reviewed merely described the voting system used, without justifying its selection.

As noted above, characteristics of voting systems that may be crucial weaknesses in one situation may be irrelevant in another. Although comprehensive analysis of the strengths and weaknesses of different voting systems is complex (Elkind et al. 2011; Nurmi 2012), it is possible to compare the behavior of candidate systems in particular circumstances using simulations (Gavish & Gerdes 1997). People designing or choosing voting systems might use simulations to anticipate potential manipulations and implement measures to mitigate them, if such manipulative voting strategies are judged unfair.

Voting systems embody different philosophies. For example, winners under Condorcet voting can defeat every other alternative in pairwise contests. They almost always win under approval voting. However, under preferential voting, they often lose because they split the vote with one or more moderate alternatives. A Condorcet winner may be defeated through manipulation by individual voters or coalitions of voters. Approval voting, however, ensures the election of a Condorcet winner, encouraging sincere voting (Brams & Sanver 2006).

Preferential voting encourages honest support for alternatives that are unlikely to succeed. In simple majority voting, in contrast, voters may decide against voting for their preferred candidate if they are unlikely to win, believing their vote would be effectively wasted. Instead, they may vote for a generally more favored alternative, rather than risk a much less preferred alternative being selected. That is, people may use knowledge about the preferences of other voters to make strategic voting decisions. Preferential voting takes the full order of preferences into account. If a person’s first preference is eliminated early, their vote is reallocated to their second preference. People do not have to anticipate the favored alternatives to be sure their preferences will count in determining the final outcome (Brams 2004).

The myriad properties and sensitivities of alternative voting systems raise the important question, what are the qualities of a desirable outcome? Do we prefer to come away with everyone moderately pleased, or some people very pleased and others disgruntled? As previously noted, every voting system has at least one flaw. Many people, including those engaged in environmental decision making, trust voting systems to aggregate preferences fairly. Yet, most people are unaware that the outcome of voting may be determined by interactions between properties of the system and strategic behavior of other voters. Every voting system may seem unfair, at least in some circumstances. The people responsible for designing voting systems should be explicit about their choice, the possibilities of counterintuitive outcomes, and its susceptibility to strategic manipulation.

If one were to abandon voting, the only realistic option would be to ask for more information, that is, cardinal, rather than ordinal, preferences, expressing how much one alternative is preferred over another. Measures of individual benefit, equity, or efficiency of social welfare avoid many of the difficulties that arise in voting systems (Sen 1999). For example, Chakravarty and Kaplan (2010) outline circumstances in which it may be preferable to allow people to express how much they care about an alternative and to have the strength of the preference contribute to the outcome. One may elicit cardinal preferences and combine them in a number of ways. However, it is unclear to what extent such systems satisfy a range of relevant, desirable criteria equivalent to those that apply to voting systems (Nurmi 2012). And as noted above, they may be costly and time consuming. In a rare example, Phillips et al. (2010) considered a case like that outlined in Table10 and evaluated a suite of quantitative systems against a set of desirable properties. Many more such studies are required.

For environmental decisions involving multiple stakeholders, voting systems can be effective tools for policy makers to generate group decisions from individual preferences. It is clear from this review that the choice of voting system involves social judgments about what sort of voting system is most fair. Participants in environmental decision making groups should have the opportunity to scrutinize alternative voting systems before one is chosen.
